# Vitamin D3 pretreatment regulates renal inflammatory responses during lipopolysaccharide-induced acute kidney injury

**DOI:** 10.1038/srep18687

**Published:** 2015-12-22

**Authors:** Shen Xu, Yuan-Hua Chen, Zhu-Xia Tan, Dong-Dong Xie, Cheng Zhang, Zhi-Hui Zhang, Hua Wang, Hui Zhao, De-Xin Yu, De-Xiang Xu

**Affiliations:** 1 The Second Affiliated Hospital, Anhui Medical University, Hefei, China; 2 Department of Toxicology, Anhui Medical University, Hefei, China; 3 Department of Histology and Embryology, Anhui Medical University, Hefei, China

## Abstract

Vitamin D receptor (VDR) is highly expressed in human and mouse kidneys. Nevertheless, its functions remain obscure. This study investigated the effects of vitamin D3 (VitD3) pretreatment on renal inflammation during lipopolysaccharide (LPS)-induced acute kidney injury. Mice were intraperitoneally injected with LPS. In VitD3 + LPS group, mice were pretreated with VitD3 (25 μg/kg) at 48, 24 and 1 h before LPS injection. As expected, an obvious reduction of renal function and pathological damage was observed in LPS-treated mice. VitD3 pretreatment significantly alleviated LPS-induced reduction of renal function and pathological damage. Moreover, VitD3 pretreatment attenuated LPS-induced renal inflammatory cytokines, chemokines and adhesion molecules. In addition, pretreatment with 1,25(OH)2D3, the active form of VitD3, alleviated LPS-induced up-regulation of inflammatory cytokines and chemokines in human HK-2 cells, a renal tubular epithelial cell line, in a VDR-dependent manner. Further analysis showed that VitD3, which activated renal VDR, specifically repressed LPS-induced nuclear translocation of nuclear factor kappa B (NF-κB) p65 subunit in the renal tubules. LPS, which activated renal NF-κB, reciprocally suppressed renal VDR and its target gene. Moreover, VitD3 reinforced the physical interaction between renal VDR and NF-κB p65 subunit. These results provide a mechanistic explanation for VitD3-mediated anti-inflammatory activity during LPS-induced acute kidney injury.

Sepsis is a severely deregulated inflammatory response to infection characterized by a systemic inflammatory state and multiple organ failure^1^,^2^. Acute kidney injury, defined as a rapid renal dysfunction with severe tubular damage, is a frequent and serious complication of sepsis in intensive care unit (ICU) patients with an extremely high mortality^3^,^4^,^5^. It is increasingly recognized that sepsis is the most important cause of acute kidney injury in critically ill patients, account for 50% or more of acute kidney injury in ICUs^6^. Lipopolysaccharide (LPS), a component of the outer membrane in Gram-negative bacteria, is involved in the pathogenesis of sepsis-induced acute kidney injury^7^,^8^. Thus, LPS has been widely used as a model of experimental sepsis-induced acute kidney injury^9^. As there has been no effective treatment for acute kidney injury, novel preventive and therapeutic interventions are urgently needed to tackle sepsis-induced acute kidney injury.

Increasing evidence demonstrates that inflammatory cytokines, such as tumor necrosis factor alpha (TNF-α), contribute to the development of sepsis-induced acute kidney injury^10^,^11^. Prophylactic TNF-α antibody and pentoxifylline, an inhibitor of TNF-α synthesis, protected mice from sepsis-induced acute kidney injury^12^,^13^. Arachidonic acid, which is transformed by cyclooxygenase (COX)-1 and COX-2 into prostaglandins, prostacyclins and thromboxanes, plays a critical role in sepsis-induced acute kidney injury. An earlier study showed that thromboxane (Tx)A_2_ receptor knockout or a selective TxA_2_ receptor antagonist alleviated LPS-induced renal vasoconstriction and acute renal injury in mice^14^. A recent study found that netrin-1 suppressed ischemic acute kidney injury through inhibiting COX-2-mediated production of prostaglandin E2 and thromboxanes^15^.

Vitamin D is known for its classical functions in calcium uptake and bone metabolism^16^. Recently, vitamin D is recognized for its non-classical actions including the modulation of innate immune and the regulation of cell proliferation^17^,^18^. Vitamin D itself is devoid of biological activity. Vitamin D3 (VitD3) is converted to 25(OH)D3 by cytochrome P450 (CYP)2R1 in the liver^19^. 25(OH)D3 is then converted into 1,25(OH)2D3 (also known as calcitriol), the active form of VitD3, by CYP27B1 in the kidney^20^. The actions of VitD3 are mediated by vitamin D receptor (VDR) that binds 1,25(OH)2D3 to induce both transcriptional and non-genomic responses^21^. Indeed, all components that mediate vitamin D activity, such as VDR and CYP27B1, are highly expressed in human and mouse kidneys^22^,^23^. Several studies demonstrated that VitD3 suppressed TGF-β-mediated tubular epithelial-to-mesenchymal transition and renal fibrosis in a VDR-dependent manner^24^,^25^. According to a recent report, the activated VDR physically interacts with IκB kinase β (IKKβ) to block TNF-α-mediated translocation of nuclear factor kappa B (NF-κB) p65 subunit from cytoplasm to nuclei in HEK293 cell^26^.

The aim of the present study was to investigate the effects of VitD3 pretreatment on early inflammatory response during LPS-induced acute kidney injury. We showed that VitD3 pretreatment alleviated early inflammatory response in LPS-induced acute kidney injury. We demonstrate that there is a mutual repression between VitD3-activated renal VDR signaling and LPS-activated renal NF-κB signaling. The interaction between renal VDR and NF-κB p65 provides a mechanistic explanation for VitD3-mediated anti-inflammatory activity during LPS-induced acute kidney injury.

## Results

### VitD3 pretreatment alleviates LPS-induced acute kidney injury in mice

**Figure 1:**
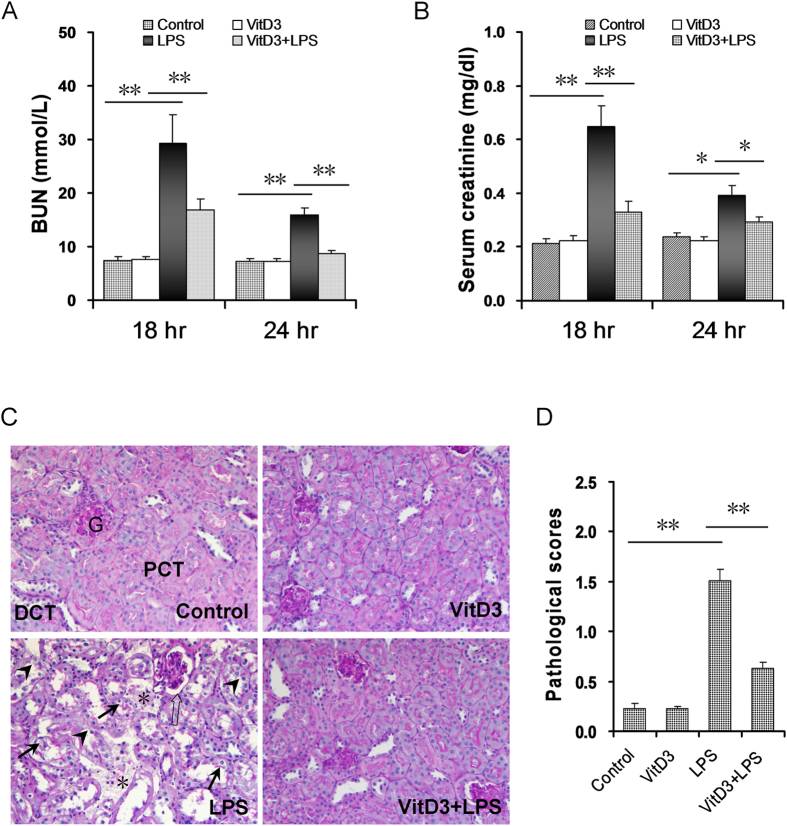
VitD3 pretreatment alleviates on LPS-induced acute kidney injury. In LPS group, mice were i.p. injected with LPS (1.0 mg/kg). In VitD3 + LPS group, mice were pretreated with three doses of VitD3 (25 μg/kg) at 48, 24 and 1 h before LPS injection. Kidney and blood samples were collected at 18 h and 24 h after LPS injection. (**A,B**) Renal function was measured 18 h and 24 h after LPS injection. (**A**) BUN; (**B**) Serum creatinine. (**C,D**) Renal histopathology was evaluated 18 h after LPS injection. (**C**) Representative PAS staining of renal cortex. An obvious renal pathological damage, including edema of renal tubular epithelial cells (solid arrows), dilation of renal capsule cavity (hollow arrows), the loss of tubular brush borders (arrowheads), destruction of tubular structures and the epithelial cells of the local focal necrosis collapse (*), was observed 18 h after LPS. DCT: distal convoluted tubule; PCT: proximal convoluted tubule; G: glomerulus. (**D**) Pathological scores of tubular damage among different groups. All data were expressed as means ± S.E.M (n = 10). **P < 0.01, *P < 0.05.

The effects of VitD3 pretreatment on LPS-induced renal function were analyzed. As shown in Fig. 1A, the level of BUN was markedly increased 18 h and 24 h after LPS injection. Correspondingly, the level of serum creatinine was elevated 18 h and 24 h after LPS injection (Fig. 1B). Moreover, the levels of BUN and serum creatinine were higher at 18 h after LPS injection than at 24 h after LPS injection (Fig. 1A,B). Interestingly, VitD3 pretreatment protected against LPS-induced impairment of renal function (Fig. 1A,B). The effects of VitD3 pretreatment on LPS-induced renal pathological damage were then analyzed. As expected, an obvious renal pathological damage, including edema of renal tubular epithelial cells, dilation of renal capsule cavity, destruction of tubular structures, the epithelial cells of the local focal necrosis collapse and loss of tubular brush borders, was observed 18 h after LPS injection (Fig. 1C). VitD3 pretreatment obviously attenuated LPS-induced pathological damage in the kidneys (Fig. 1C,D).

### Effects of VitD3 pretreatment on LPS-induced renal inflammatory cytokines, chemokines, *icam-1*, *vcam-1* and *cox-2*

**Figure 2:**
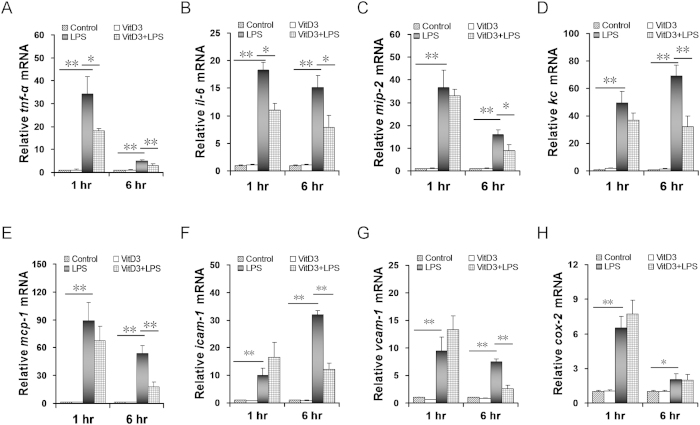
Effects of VitD3 pretreatment on LPS-induced renal inflammatory cytokines, chemokines, *cox-2*, *icam-1* and *vcam-1*. In LPS group, mice were i.p. injected with LPS (1.0 mg/kg). In VitD3 + LPS group, mice were pretreated with three doses of VitD3 (25 μg/kg) at 48, 24 and 1 h before LPS injection. Kidneys were collected either 1 or 6 h after LPS injection. Inflammatory cytokines, chemokines, *cox-2*, *icam-1* and *vcam-1* were measured using real-time RT-PCR. (**A**) *tnf-α*; (**B**) *il-6*; (**C**) *mip-2*; (**D**) *kc*; (**E**) *mcp-1*; (**F**) *icam-1*; (**G**) *vcam-1*; (**H**) *cox-2*. All data were expressed as means ± S.E.M (n = 10). *P < 0.05, **P < 0.01.

The effects of VitD3 pretreatment on LPS-induced renal inflammatory cytokines were analyzed. As expected, renal *tnf-α* and *il-6*, two inflammatory cytokine genes, were markedly up-regulated 1 h after LPS injection and remained increased 6 h after LPS injection. Interestingly, VitD3 pretreatment significantly attenuated LPS-induced up-regulation of renal inflammatory cytokines (Fig. 2A,B). The effects of VitD3 pretreatment on LPS-induced renal chemokines were then analyzed. As expected, renal *mip-2*, *kc* and *mcp-1*, three chemokine genes, were markedly up-regulated 1 h after LPS injection and remained increased 6 h after LPS injection (Fig. 2C–E). Although VitD3 pretreatment had little effect on renal chemokines at 1 h after LPS, the expression of renal chemokines at 6 h after LPS injection was significantly repressed in VitD3-pretreated mice (Fig. 2C–E). Next, the effects of VitD3 pretreatment on LPS-induced renal *icam-1* and *vcam-1* expression were analyzed. As expected, renal *icam-1* and *vcam-1* mRNAs were markedly up-regulated 1 h after LPS injection and remained increased 6 h after LPS injection (Fig. 2F,G). Although VitD3 pretreatment had little effect on the expression of renal *icam-1* and *vcam-1* mRNAs at 1 h after LPS injection, the expression of renal *icam-1* and *vcam-1* mRNAs at 6 h after LPS injection was significantly repressed in VitD3-pretreated mice (Fig. 2F,G). Finally, the effects of VitD3 pretreatment on LPS-induced renal *cox-2* were analyzed. As shown in Fig. 2H, renal *cox-2* mRNA was markedly up-regulated at 1 h after LPS injection and remained increased at 6 h after LPS injection. VitD3 alone had no effect on the expression of renal *cox-2*. Moreover, VitD3 pretreatment had little effect on LPS-induced upregulation of renal *cox-2* (Fig. 2H).

### Effects of 1,25(OH)2D3 on LPS-evoked inflammatory cytokines and chemokines in human HK-2 cells

**Figure 3:**
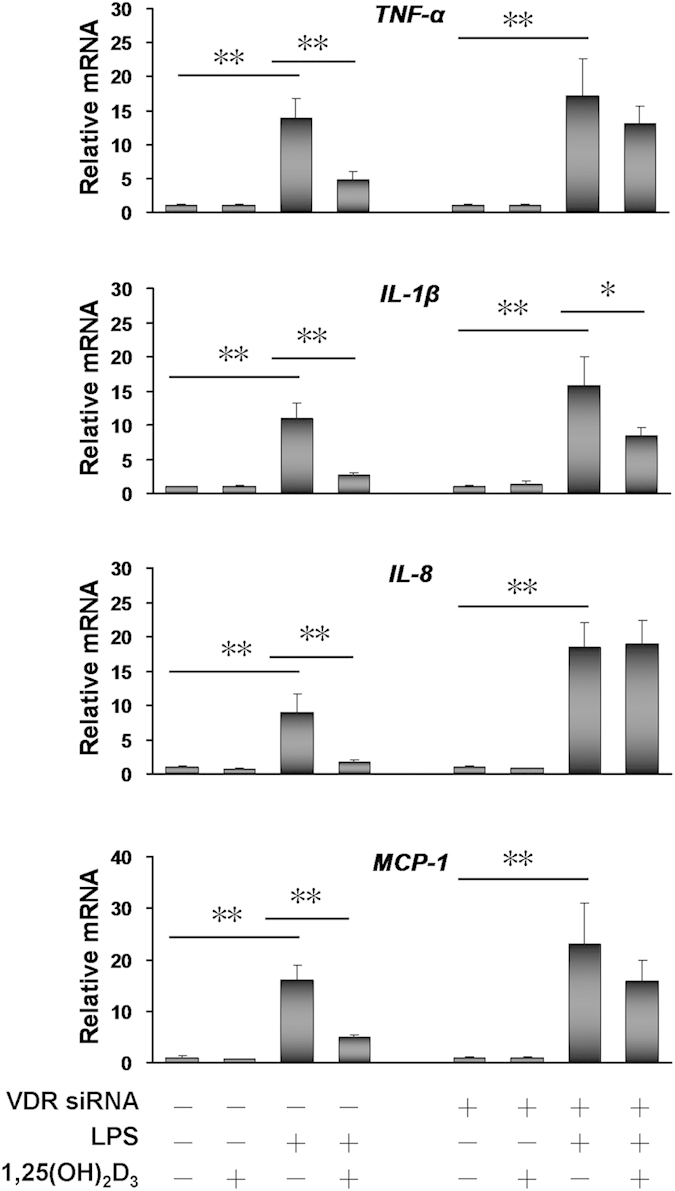
Effects of 1,25(OH)2D3 on LPS-induced inflammatory cytokines and chemokines in human HK-2 cells. Human HK-2 cells were transfected with either pooled VDR siRNA (100 nM) or random siRNA (100 nM, as control) as Materials and Methods. Human HK-2 cells were then cultured with LPS (5.0 μg/ml) for 2 h in presence or absence of 1,25-(OH)2D3 (100 nM). *TNF-α*, *IL-1β*, *IL-8* and *MCP-1* mRNAs were measured using real-time RT-PCR. VDR siRNA (–) denotes the pretreatment with random siRNA as control. All data were expressed as means ± S.E.M (n = 6). *P < 0.05, **P < 0.01.

To further explore the functional role of VDR in modulating LPS-induced inflammatory cytokines and chemokines, VDR-siRNA was used to inhibit the expression of VDR in human HK-2 cells. As expected, VDR mRNA was down-regulated by 80% in VDR-siRNA-transfected human HK-2 cells (Supplementary Fig. 1). The inflammatory cytokines and chemokines were measured in LPS-stimulated random siRNA (as control)-transfected and VDR-siRNA-transfected human HK-2 cells. As expected, *TNF-α*, *IL-1β*, *IL-8* and *MCP-1* mRNAs were obviously upregulated in LPS-stimulated random siRNA-transfected and VDR-siRNA-transfected human HK-2 cells (Fig. 3). We then compared the effects of 1,25(OH)2D3 on LPS-evoked inflammatory cytokines and chemokines in random siRNA-transfected and VDR-siRNA-transfected human HK-2 cells. As expected, LPS-evoked up-regulation of *TNF-α*, *IL-1β*, *IL-8* and *MCP-1* was markedly attenuated by 1,25-(OH)2D3 pretreatment in random siRNA-transfected human HK-2 cells. Although LPS-evoked up-regulation of *IL-1β* was slightly attenuated by 1,25-(OH)2D3, 1,25-(OH)2D3 pretreatment had little effect on LPS-evoked up-regulation of *TNF-α*, *IL-8* and *MCP-1* in VDR-siRNA-transfected human HK-2 cells (Fig. 3).

### Effects of VitD3 pretreatment on LPS-activated renal MAPK p38 and PI3K/Akt signaling

**Figure 4:**
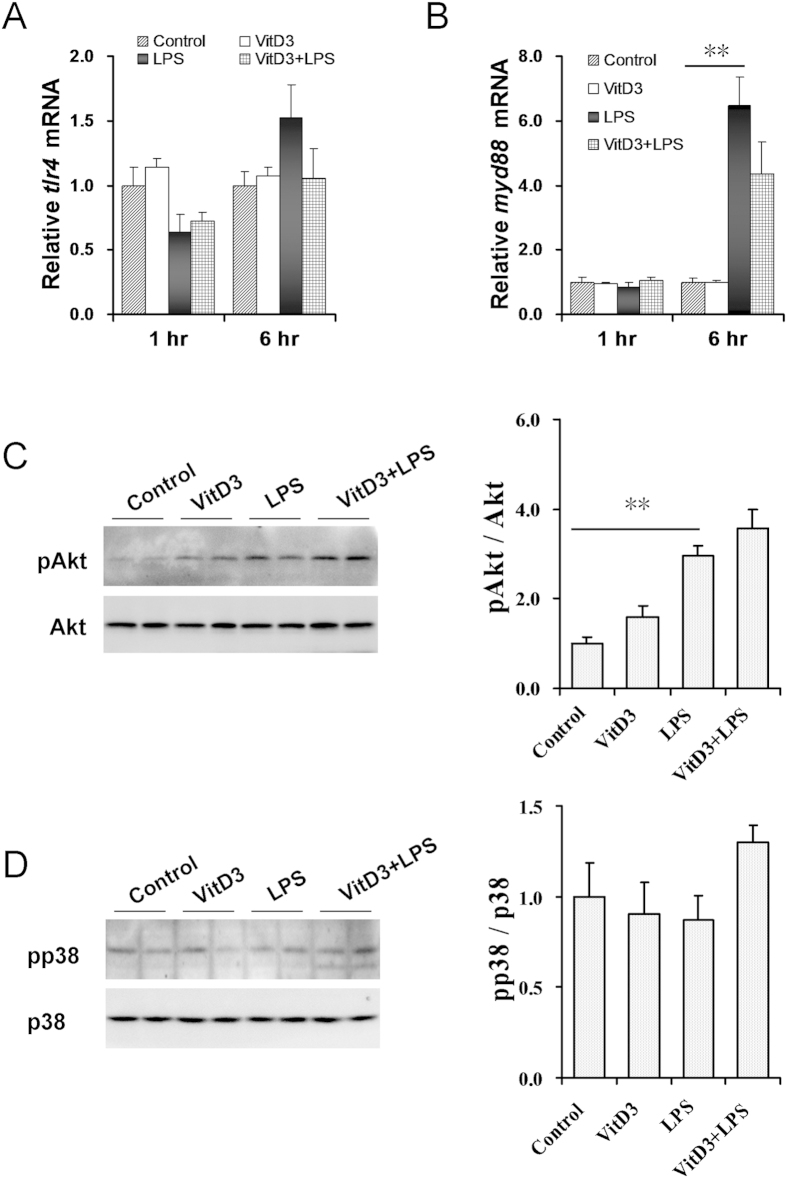
Effects of VitD3 pretreatment on renal MAPK p38 and PI3K/Akt signaling in LPS-induced acute kidney injury. In LPS group, mice were i.p. injected with LPS (1.0 mg/kg). In VitD3 + LPS group, mice were pretreated with three doses of VitD3 (25 μg/kg) at 48, 24 and 1 h before LPS injection. Kidneys were collected 1 h after LPS injection. (**A,B**) Renal *tlr4* and *myd88* mRNAs were measured using real-time RT-PCR. (**A**) *tlr4*. (**B**) *myd88*. (**C,D**) Renal pAkt and pp38 were measured using immunoblots. (**C**) pAkt. A representative gel for pAkt (upper panel) and Akt (lower panel) was shown. (**D**) pp38. A representative gel for pp38 (upper panel) and p38 (lower panel) was shown. All experiments were duplicated for four times. All data were expressed as means ± S.E.M. (n = 8). **P < 0.01.

As shown in Fig. 4A, no significant difference on renal *tlr4* mRNA was observed among different groups. Moreover, VitD3 alone did not affect renal *myd88* expression (Fig. 4B). As expected, renal *myd88* mRNA was up-regulated 6 h after LPS injection. Interestingly, VitD3 pretreatment had little effect on LPS-induced upregulation of renal *myd88* (Fig. 4B). The effects of VitD3 pretreatment on LPS-induced renal Akt and MAPK p38 phosphorylation were analyzed. As shown in Fig. 4C, the level of renal phosphorylated Akt was obviously elevated 1 h after LPS injection. By contrast, the level of renal phosphorylated p38 was not elevated 1 h after LPS injection (Fig. 4D), indicating that renal MAPK p38 signaling is not activated 1 h after LPS injection. Unexpectedly, VitD3 pretreatment had little effect on LPS-induced renal Akt phosphorylation (Fig. 4C).

### Effects of VitD3 pretreatment on LPS-activated renal NF-kB signaling

**Figure 5:**
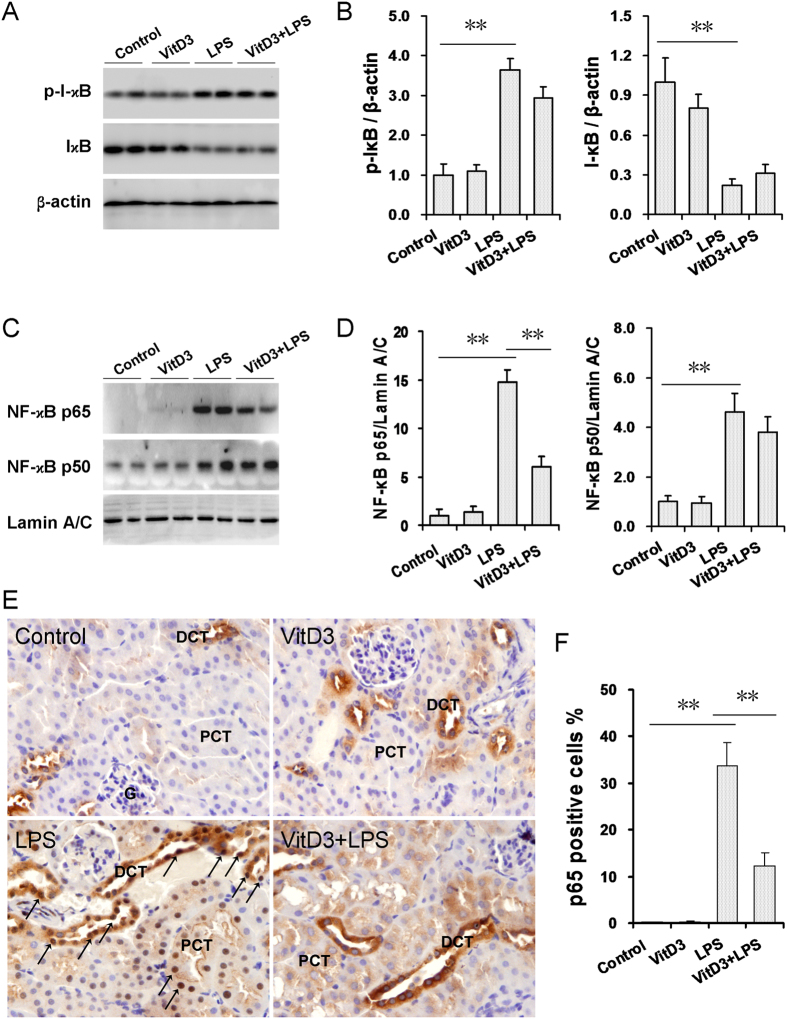
Effects of VitD3 pretreatment on LPS-activated renal NF-kB signaling. In LPS group, mice were i.p. injected with LPS (1.0 mg/kg). In VitD3 + LPS group, mice were pretreated with three doses of VitD3 (25 μg/kg) at 48, 24 and 1 h before LPS injection. Kidneys were collected 1 h after LPS injection. (**A,B**) Renal I-κB and p-IκB were measured using immunoblots. All experiments were duplicated for four times. (**A**) A representative gel for p-IκB (upper panel), I-κB (middle) and β-actin (lower panel) was shown. (**B**) p-IκB/β-actin and I-κB/β-actin. All data were expressed as means ± S.E.M. (n = 8). **P < 0.01. (**C,D**) Renal nuclear NF-κB p65 and p50 subunits were measured using immunoblots. All experiments were duplicated for four times. (**C**) A representative gel for NF-κB p65 (upper panel), p50 (middle) and lamin A/C (lower panel) was shown. (**D**) p65/ lamin A/C and p50/ lamin A/C. All data were expressed as means ± S.E.M. (n = 8). **P < 0.01. (**E**) Renal nuclear NF-kB p65 translocation was analyzed using IHC. Representative photomicrographs of renal histological specimens from mice treated with NS, VitD3, LPS (1H) and VitD3 + LPS are shown. Original magnification × 400. Nuclear NF-kB p65 translocation was mainly observed in the nuclei of the distal convoluted tubules (arrows). DCT: distal convoluted tubule; PCT: proximal convoluted tubule; G: glomerulus. (**F**) Percent of p65 positive cells among different groups. All data were expressed as means ± S.E.M (n = 10). **P < 0.01.

A recent study demonstrated that renal NF-κB signaling was involved in the pathogenesis of LPS-induced acute kidney injury^27^. The effects of VitD3 pretreatment on LPS-activated renal NF-κB signaling were analyzed. As expected, renal phosphorylated IκB level was significantly increased in LPS-treated mice (Fig. 5A,B). Correspondingly, renal I-κB level was significantly decreased in LPS-treated mice (Fig. 5A,B). By contrast, the levels of nuclear NF-κB p65 and p50 subunits in the kidneys were markedly elevated 1 h after LPS injection (Fig. 5C,D), indicating that LPS could rapidly evoke renal NF-κB p65 and p50 translocation from cytoplasm to nuclei. Immunohistochemistry observed a strong immunoreactivity in the nuclei of the distal convoluted tubules (DCT) and a relatively weak immunoreactivity in the nuclei of the proximal convoluted tubules (PCT) (Fig. 5E). These results suggest that LPS-evoked nuclear translocation of renal NF-kB p65 subunit was mainly distributed in the DCT, and to a lesser extent, in the PCT. Of interest, VitD3 pretreatment had little effect on LPS-induced I-κB phosphorylation. Moreover, VitD3 pretreatment did not inhibit LPS-induced reduction of renal IκBα level (Fig. 5A,B). In addition, VitD3 pretreatment did not inhibit LPS-induced translocation of nuclear NF-κB p50 subunit (Fig. 5C,D). Instead, VitD3 pretreatment blocked LPS-induced translocation of NF-κB p65 subunit from the cytoplasm to the nuclei in the convoluted tubules (Fig. 5C–F).

### Effects of LPS on VitD3-activated renal VDR signaling

**Figure 6:**
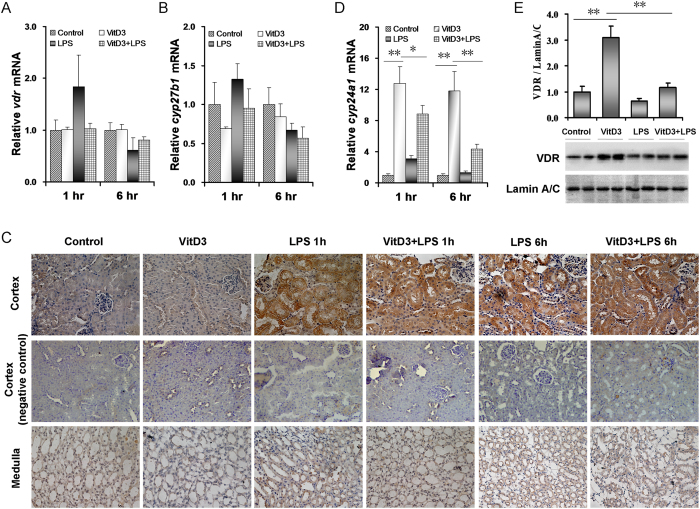
Effects of LPS on VitD3-activated renal VDR signaling. In LPS group, mice were i.p. injected with LPS (1.0 mg/kg). In VitD3 + LPS group, mice were pretreated with three doses of VitD3 (25 μg/kg) at 48, 24 and 1 h before LPS injection. Kidneys were collected either 1 or 6 h after LPS injection. (**A,B**) Renal *vdr* and *cyp27b1* mRNAs were measured using real-time RT-PCR. (**A**) *vdr*. (**B**) *cyp27b1*. (**C**) Renal CYP27B1 was analyzed using IHC. Representative photomicrographs of renal histological specimens are shown. (**D**) Renal *cyp24a1* mRNAs were measured using real-time RT-PCR. (**E**) Nuclear VDR was measured using immunoblots. All experiments were duplicated for four times. A representative gel for VDR (upper panel) and lamin A/C (lower panel) was shown. All data were expressed as means ± S.E.M. (n = 8). *P < 0.05, **P < 0.01.

The effects of LPS on VitD3-activated renal VDR signaling were analyzed. As shown in Fig. 6A, VitD3 alone had no effect on renal *vdr* expression. Interestingly, renal *vdr* mRNA was slightly upregulated at 1 h after LPS injection and then downregulated at 6 h after LPS injection. The effects of LPS on renal *cyp27b1*, an enzyme gene that transformed 25(OH)D3 into 1,25(OH)2D3, were analyzed. Although no significant difference on renal *cyp27b1* mRNA was observed among different groups (Fig. 6B), the level of CYP27B1 protein in proximal tubule of renal cortex was markedly elevated at 1 h after LPS injection and remained increased at 6 after LPS injection (Fig. 6C). Interestingly, LPS had no effect on the level of CYP27B1 protein in renal medulla (Fig. 6C). The effects of LPS on renal *cyp24a1*, a target gene of VDR signaling, are presented in Fig. 6D. As expected, renal *cyp24a1* mRNA was upregulated by more than 12 folds in VitD3-pretreated mice. Interestingly, VitD3-induced upregulation of renal *cyp24a1* was obviously inhibited at 1 h after LPS injection and remained repressed at 6 h after LPS injection (Fig. 6D). Finally, the effects of LPS on renal nuclear VDR were analyzed. As expected, nuclear VDR level in the kidneys was significantly elevated in VitD3-pretreated mice (Fig. 6E), indicating that VitD3 pretreatment promotes renal VDR translocation from the cytoplasm to the nucleus. LPS significantly inhibited VitD3-evoked nuclear VDR translocation in the kidneys (Fig. 6E).

### The interaction between renal VDR and NF-κB p65

**Figure 7:**
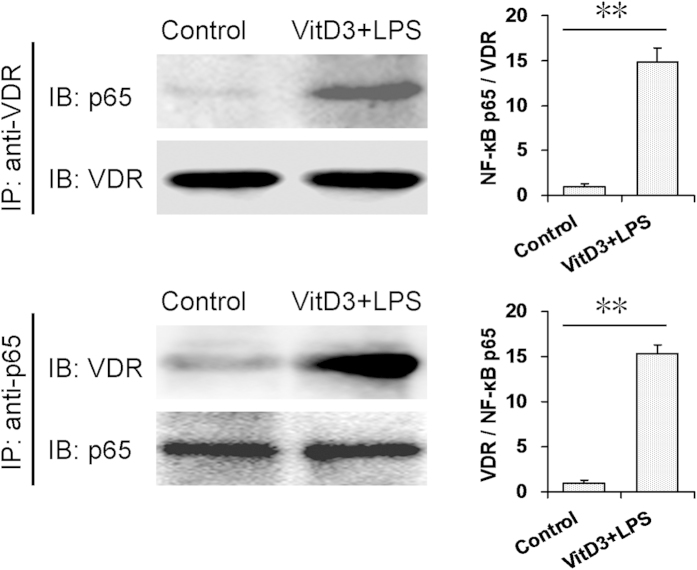
The interaction between renal VDR and NF-κB p65. In VitD3 + LPS group, mice were pretreated with three doses of VitD3 (25 μg/kg) at 48, 24 and 1 h before LPS (1.0 mg/kg) injection. Kidney samples were collected 1 h after LPS injection. The nuclear fractions were prepared from kidneys and incubated with agarose-conjugated either VDR or NF-κB p65 antibody. NF-κB p65 and VDR were measured using immunoblots. All experiments were duplicated for four times. All data were expressed as means ± S.E.M. (n = 4). **P < 0.01.

The interaction between renal VDR and NF-κB p65 was determined by CoIP. As expected, VitD3 plus LPS treatments increased the level of NF-κB p65 in the immunocomplexes precipitated by anti-VDR antibody (Fig. 7). Correspondingly, VitD3 plus LPS treatments increased the level of VDR in the immunocomplexes precipitated by anti-p65 antibody (Fig. 7). These results suggest that VitD3 pretreatment reinforces the interaction between VDR and NF-κB p65 in the kidneys.

## Discussion

An earlier study indicated that paricalcitol, a vitamin D analog, prevented rats from cisplatin-induced acute renal injury by suppressing apoptosis and proliferation^28^. According to a recent report, paricalcitol protected against ischemia/reperfusion-induced acute kidney injury by suppressing TLR4-NF-κB mediated inflammation^29^. The present study investigated the effect of VitD3 pretreatment on LPS-induced acute kidney injury. We showed that VitD3 prevented LPS-induced reduction of renal function. In addition, VitD3 pretreatment obviously alleviated LPS-induced pathological damage in the kidneys. These results suggest that VitD3 pretreatment protects mice from sepsis-induced acute kidney injury.

It is increasingly recognized that inflammatory cytokines, such as TNF-α, are the major mediators of sepsis-induced acute kidney injury^10^,^11^. MCP-1 and IL-8, two chemokines, play key roles in the recruitment of inflammatory cells into renal interstitium in sepsis-induced acute kidney injury^30^. The present study showed that renal *tnf-α* and *il-6* were rapidly up-regulated after LPS injection. Moreover, renal *mcp-1* mRNA was markedly elevated after LPS injection. In addition, renal *mip-2* and *kc*, two functional analogues of human IL-8, were obviously up-regulated at 1 and 6 h after LPS injection. Interestingly, LPS-induced up-regulation of renal *tnf-α* and *il-6* was significantly attenuated by VitD3. Moreover, LPS-induced up-regulation of renal *mcp-1, mip-2* and *kc* was obviously repressed in VitD3-pretreated mice. The role of adhesion molecules, such as ICAM-1 and VCAM-1, in LPS-induced acute kidney injury is well established^7^,^31^. Indeed, the present study showed that the expression of renal *icam-1* and *vcam-1* was up-regulated after LPS injection. Interestingly, LPS-induced up-regulation of renal *icam-1* and *vcam-1* was markedly attenuated by VitD3 pretreatment. These results suggest that VitD3 pretreatment inhibits early inflammatory response during LPS-induced acute kidney injury.

It is well known that both adaptive and innate immune cells express VDR^17^. Indeed, immune cells play a predominant role in the pathogenesis of sepsis. To demonstrate whether renal VDR also plays an important role in VitD3-mediated anti-inflammatory effect, the effects of 1,25(OH)2D3, an active form of VitD3, on LPS-evoked inflammatory cytokines and chemokines in human HK-2 cells, a renal tubular epithelial cell line, were analyzed. As expected, *TNF-α* and *IL-1β*, two inflammatory cytokines, and *IL-8* and *MCP-1*, two chemokines, were markedly upregulated in LPS-treated human HK-2 cells. Moreover, pretreatment with 1,25(OH)2D3 significantly attenuated LPS-evoked inflammatory cytokines and chemokines in human HK-2 cells. To further determine the functional role of renal VDR in modulating LPS-induced inflammatory cytokines and chemokines, siRNA was used to inhibit VDR expression in human HK-2 cells. As expected, *TNF-α*, *IL-1β*, *IL-8* and *MCP-1* were obviously up-regulated in LPS-stimulated VDR-siRNA-transfected human HK-2 cells. Interestingly, 1,25-(OH)2D3 had little effect on LPS-induced inflammatory cytokines and chemokines in VDR-siRNA-transfected human HK-2 cells. These results suggest that renal VDR is involved in the regulation of early renal inflammatory responses during LPS-induced acute kidney injury. The present study does not exclude the role of VDR in both adaptive and innate immune cells on VitD3-mediated anti-inflammatory effect in LPS-induced acute kidney injury. Thus, additional work is required to determine the role of immune cells in VitD3-mediated protection against LPS-induced acute kidney injury.

The mechanism through which VDR plays its anti-inflammatory activity remains with debate. According to an earlier report, 1,25(OH)2D3, an active VitD3 that activates VDR signaling, stimulates MAPK p38 phosphorylation in aortic smooth muscle cells^32^. A recent study showed that 1,25(OH)2D3 activated MAPK p38 and PI3K/Akt signaling in cultured endothelial cells^33^. However, two recent studies found that 1,25(OH)2D3 obviously down-regulated LPS-evoked inflammatory cytokines through inhibiting MAPK p38 and Akt phosphorylation in macrophages^34^,^35^. The present study investigated the effects of VitD3 pretreatment on renal inflammatory signaling in LPS-induced acute kidney injury. Although renal MAPK p38 was not activated 1 h after LPS injection, renal inflammatory cytokines were significantly up-regulated, indicating that LPS-induced upregulation of inflammatory cytokines is independent of renal MAPK p38 signaling. Interestingly, the level of renal phosphorylated Akt was significantly elevated in LPS-treated mice. Moreover, renal NF-κB was activated 1 h after LPS injection, as determined by the reduced I-κB level and the elevated nuclear NF-κB p65 and p50 levels. Unexpectedly, VitD3 pretreatment did not repress LPS-induced renal Akt phosphorylation. By contrary, VitD3 inhibited LPS-induced renal NF-κB activation. These results suggest that VitD3 down-regulates renal inflammatory cytokines by inhibiting renal NF-κB signaling.

According to a recent report using GST pull-down assay, the activated VDR physically interacts with IκB kinase β (IKKβ), abrogates IKKβ phosphorylation and abolishes IKK to phosphorylate I-κB, thus prevents nuclear translocation of NF-κB p65/p50 subunits^26^. The present study analyzed the effects of VitD3 pretreatment on LPS-evoked renal I-κB phosphorylation. Unexpectedly, VitD3 pretreatment had little effect on LPS-evoked renal I-κB phosphorylation. Correspondingly, VitD3 pretreatment did not inhibit LPS-induced reduction of I-κB. Moreover, VitD3 pretreatment did not inhibit LPS-induced translocation of nuclear NF-κB p50 subunit. Instead, VitD3 pretreatment blocked LPS-induced translocation of NF-κB p65 subunit from the cytoplasm to the nuclei in the renal tubules. Several studies have demonstrated that the activated nuclear receptors, such as pregnane X receptor and liver X receptor, repress NF-κB signaling in macrophages^36^,^37^. Conversely, LPS-activated NF-κB inhibits nuclear receptor signaling in hepatocytes and intestinal epithelial cells^38^,^39^. Indeed, VDR, also a nuclear receptor, is highly expressed in tubular epithelial cells of human and rodent kidneys^23^,^40^. The present study hypothesizes that the activated VDR inhibits LPS-activated renal NF-κB signaling through its interaction with NF-κB p65 subunit. Following evidence demonstrates the hypothesis that VitD3 inhibits renal NF-κB signaling through the interation between VDR and NF-κB p65 subuni. First, VitD3, which activated renal VDR, simultaneously blocked LPS-evoked nuclear NF-κB p65 translocation and repressed its downstream target genes in the kidneys. Second, LPS, which activated renal NF-κB, simultaneously abolished VitD3-evoked nuclear VDR translocation and inhibited its downstream target genes in the kidneys. Third, LPS-evoked nuclear NF-kB p65 translocation was mainly observed in the nuclei of the distal convoluted tubules, and to a lesser extent, in the nuclei of the proximal convoluted tubules. Correspondingly, VDR was highly expressed in the distal convoluted tubules, and to a relatively lower level, in the proximal convoluted tubules^23^. To elucidate the mechanism through which VitD3-activated VDR inhibits LPS-evoked nuclear NF-κB p65 translocation in the kidneys, CoIP was used to test physical association between renal VDR and NF-κB subnuits in LPS-induced acute kidney injury. As expected, VitD3 pretreatment reinforced the interaction between renal VDR and NF-κB p65 during LPS-induced acute kidney injury. Taken together, these results suggest that VitD3-activated VDR inhibits LPS-evoked renal NF-κB activation through its interaction with NF-κB p65 subunit.

Renal VDR as a regulator of inflammatory response may have preventive and therapeutic implications. Several studies showed that vitamin D status was negatively associated with acute kidney injury in critically ill patients^41^,^42^. According to a recent report, vitamin D deficiency aggravated the progression of chronic kidney disease after ischemia/reperfusion -induced acute kidney injury^43^. The present study showed that VitD3 pretreatment protected against LPS-induced acute kidney injury by regulating early renal inflammatory responses. Therefore, VitD3 may be used as a potential protective agent for clinical prevention and therapy especially in high-risk situations in which the patients are infected with bacteria.

In summary, the present study investigated the effects of VitD3 pretreatment on early renal inflammatory response during LPS-induced acute kidney injury. Our results showed that VitD3 pretreatment down-regulated renal inflammatory response during LPS-induced acute kidney injury. We demonstrated for the first time that there was a mutual repression between VitD3-activated renal VDR and LPS-activated renal NF-κB. The interaction between renal VDR and NF-κB p65 subunit provides a mechanistic explanation for VitD3-mediated anti-inflammatory activity during LPS-induced acute kidney injury. Overall, the present study provides evidence for roles of renal VDR partially as an important regulator of renal inflammatory response in sepsis-induced acute kidney injury.

## Materials and Methods

### Chemicals and reagents

Lipopolysaccharide (*Escherichia coli* LPS, serotype 0127:B8), VitD3 and 1,25(OH)2D3 were purchased from Sigma Chemical Co. (St. Louis, MO). Phosphor-MAPK p38 (pp38), NF-κB p65, VDR and Lamin A/C antibodies were from Santa Cruz Biotechnologies (Santa Cruz, CA). Phosphor-Akt (pAkt), Akt and NF-κB p50 antibodies were from Cell Signaling Technology (Beverley, MA, USA). CYP27B1, inhibitor of kappa B (I-κB) and phosphor-IκB antibodies were purchased from Abcam (Cambridge, MA). Chemiluminescence (ECL) detection kit was from Pierce Biotechnology (Rockford, IL). TRI reagent was from Molecular Research Center, Inc (Cincinnati, Ohio). RNase-free DNase was from Promega Corporation (Madison, WI). All the other reagents were from Sigma or as indicated in the specified methods.

### Animals and treatments

Adult male CD-1 mice (8 week-old, 28–32 g) were purchased from Beijing Vital River whose foundation colonies were all introduced from Charles River Laboratories, Inc. The animals were allowed free access to food and water at all times and maintained on a 12-h light/dark cycle in a controlled temperature (20–25 °C) and humidity (50 ± 5%) environment. Mice were divided into four groups randomly. In LPS group, mice were intraperitoneally (i.p.) injected with a single dose of LPS (1.0 mg/kg). In control group, mice were i.p. injected with normal saline (NS). In VitD3 + LPS group, all mice were pretreated with three doses of VitD3 (each 25 μg/kg) by gavage, first dose at 48 h before LPS, second at 24 h before LPS, and third at 1 h before LPS. In VitD3 group, all mice were pretreated with three doses of VitD3 (each 25 μg/kg) by gavage at 48, 24 and 1 h before NS injection. The doses of VitD3 used in the present study referred to others^44^,^45^. Preliminary experiment showed that inflammatory cytokines were significantly up-regulated at 1 h after LPS injection and remained elevated at 6 h after LPS injection. Thus, mouse kidneys were collected 1 h and 6 h after LPS injection for the measurement of inflammatory cytokines and inflammatory signaling. Some mice were euthanized with carbon dioxide and cervical dislocation 18 h and 24 h after LPS injection. Blood samples were collected for measurement of renal function. The left kidneys were collected for histopathology. The right kidneys were collected and kept at –80 °C for subsequent experiments.

### Renal histology

Renal tissues were fixed in 4% formaldehyde and embedded in paraffin according to the standard procedure. Paraffin-embedded renal tissues were serially sectioned. At least five consecutive longitudinal sections were stained with periodic acid-schiff (PAS). Renal histopathologic alterations were evaluated using the criteria established by Solez and used previously by Conger^46^. Changes were graded on a 0 to 2 scale in a double blind fashion. There was a uniform correlation on sections from the same animal.

### Renal function

Blood samples were collected 18 h and 24 h after LPS injection. Blood urea nitrogen (BUN) was measured with colorimetric detection kits. Serum creatinine was measured with HPLC^47^.

### Cell culture and treatments

Human HK-2 cell is a renal tubular epithelial cell line, which was purchased from the American Type Cell Collection (ATCC). HK-2 cell was grown in Nunc flasks in Dulbecco’s Modified Eagle’s Medium-F12 (DMEM-F12, GIBCO) supplemented with 100 U/mL penicillin, 100 μg/mL streptomycin, 1% (v/v) insulin-transferrin-selenium, and 10% (v/v) heat-inactivated FBS in a humidified chamber with 5% CO_2_/95% air at 37 °C. HK-2 cells were seeded into 6-well culture plates at a density of 5 × 10^5^ cells/well and incubated for at least 12 hr to allow them to adhere to the plates. After washing three times with medium, human HK-2 cells were pre-incubated with 1,25-(OH)2D3 for 24 h. Human HK-2 cells were incubated with LPS (5.0 μg/ml) for 2 h in the presence or absence of 1,25-(OH)2D3 (100 nM). The concentration of 1,25-(OH)2D3 used in the present study referred to others^48^. The cells were washed with chilled PBS for three times and then harvested for real-time RT-PCR and immunoblots.

### Small interfering RNA (siRNA)

The expression of VDR levels was down-regulated in human HK-2 cells using VDR small inhibitory RNA (siRNA) using standard techniques^49^. Human VDR gene was targeted by using ON-TARGET plus SMART pool siRNA which consists of four siRNA sequences: siRNA1, 5′ GCA ACC AAG ACU ACA AGU A 3′; siRNA2, 5′ GCG CAU CAU UGC CAU ACU G 3′; siRNA3, 5′ CCA ACA CAC UGC AGA CGU A 3′; siRNA4, 5′ GCA AUG AGA UCU CCU GAC U 3′. These pooled siRNAs were used at 100 nM with standard transfection protocol using lipofectamine 2000 (Invitrogen, Carlsbad, CA). Random siRNA (100 nM) was used as a control.

### Real-time RT-PCR

Total RNA in renal tissues was extracted using TRI reagent. RNase-free DNase-treated total RNA (1.0 μg) was reverse-transcribed with AMV (Pregmega). Real-time RT-PCR was performed with a LightCycler 480 SYBR Green I kit (Roche Diagnostics GmbH) using gene-specific primers as listed in Table 1. The amplification reactions were carried out on a LightCycler 480 Instrument (Roche Diagnostics GmbH) with an initial hold step (95 °C for 5 minutes) and 50 cycles of a three-step PCR (95 °C for 15 seconds, 60 °C for 15 seconds, 72 °C for 30 seconds).

### Immunoblots

Renal lysate was prepared by homogenizing 50 mg renal tissue in 300 μl lysis buffer (50 mM Tris-HCl, pH 7.4, 150 mM NaCl, 1 mM EDTA, 1% Triton X-100, 1% sodium deoxycholate, 0.1% sodium dodecylsylphate, 1 mM phenylmethylsulfonyl fluoride) supplemented with a cocktail of protease inhibitors (Roche). For nuclear protein extraction, renal lysate was suspended in hypotonic buffer and then kept on ice for 15 min. The suspension was then mixed with detergent and centrifuged for 30 s at 14,000 × g. The nuclear pellet obtained was resuspended in complete lysis buffer in the presence of the protease inhibitor cocktail, incubated for 30 min on ice, and centrifuged for 10 min at 14,000 × g. Protein concentrations were determined with the bicinchoninic acid (BCA) protein assay reagents (Pierce, Rockford, IL, USA) according to the manufacturer’s instructions. For immunoblots, same amount of protein (40 ~ 80 μg) was separated electrophoretically by SDS-PAGE and transferred to a polyvinylidene fluoride membrane. The membranes were incubated for 2 h with the following antibodies: pAkt, Akt, pp38, p38, p-IκB, I-κB, NF-κB p65, NF-κB p50 and VDR. For total proteins, β-actin, Akt or p38 was used as a loading control. For nuclear protein, lamin A/C was used as a loading control. After washes in DPBS containing 0.05% Tween-20 four times for 10 min each, the membranes were incubated with goat anti–rabbit IgG or goat anti–mouse antibody for 2 h. The membranes were then washed for four times in DPBS containing 0.05% Tween-20 for 10 min each, followed by signal development using an ECL detection kit.

### Co-Immunoprecipitation (Co-IP)

Renal tissues were lysed with RIPA buffer (1% Nonidet P-40, 0.5% sodium deoxycholate, and 0.1% SDS in PBS, pH 7.4) containing 0.1 mM vanadyl sulfate and protease inhibitors (0.5 mg/ml aprotinin, 0.5 mg/ml trans-epoxy succinyl-L-leucylamido-(4-guanidino)butane (E-64), 0.5 mg/ml pepstatin, 0.5 mg/ml bestatin, 10 mg/ml chymostatin, and 0.1 ng/ml leupeptin). Tissue lysates (300 μg) were precleared with protein A/G-agarose and then incubated with either agarose-conjugated VDR antibody (Santa Cruz Biotechnology, Inc., Santa Cruz, CA) or NF-κB p65 antibody (Santa Cruz Biotechnology, Inc., Santa Cruz, CA) at 4 °C overnight. The precipitates were washed with cold RIPA buffer before immunoblots using either NF-κB p65 or VDR antibody.

### Immunohistochemistry (IHC)

For IHC, paraffin-embedded renal sections were deparaffinized and rehydrated in a graded ethanol series. Antigen retrieval was achieved by microwave method using sodium citrate solution with pH 6.0. After antigen retrieval and quenching of endogenous peroxidase, sections were incubated with NF-κB p65 monoclonal antibody (1:200 dilution) or CYP27B1 monoclonal antibody (1:200 dilution) at 4 °C overnight. The color reaction was developed with HRP-linked polymer detection system (Golden Bridge International, WA, USA) and counterstaining with hematoxylin^50^.

### Statistical analysis

All data were expressed as means ± SEM. SPSS 13.0 statistical software was used for statistical analysis. All statistical tests were two-sided using an alpha level of 0.05. ANOVA and the Student-Newmann-Keuls post hoc test were used to determine differences among different groups. Student *t* test was used to determine differences between two groups.

### Ethics statement

This study was approved by the Association of Laboratory Animal Sciences and the Center for Laboratory Animal Sciences at Anhui Medical University (Permit Number: 14-0017). All procedures on animals followed the guidelines for humane treatment set by the Association of Laboratory Animal Sciences and the Center for Laboratory Animal Sciences at Anhui Medical University.

## Additional Information

**How to cite this article**: Xu, S. *et al.* Vitamin D3 pretreatment regulates renal inflammatory responses during lipopolysaccharide-induced acute kidney injury. *Sci. Rep.* **5**, 18687; doi: 10.1038/srep18687 (2015).

## Supplementary Material

Supplementary Information

## Figures and Tables

**Table 1:** Oligonucleotide sequences and size of primers

Genes	Sequences	Sizes (bp)	Species
*gapdh*	Forward: 5′- ACCCCAGCAAGGACACTGAGCAAG -3′		mouse
	Reverse: 5′- GGCCCCTCCTGTTATTATGGGGGT -3′	109	
*tnf-α*	Forward: 5′- CCCTCCTGGCCAACGGCATG -3′		mouse
	Reverse: 5′- TCGGGGCAGCCTTGTCCCTT -3′	109	
*il-6*	Forward: 5′- AGACAAAGCCAGAGTCCTTCAGAGA -3′		mouse
	Reverse: 5′- GCCACTCCTTCTGTGACTCCAGC -3′	146	
*mip-2*	Forward: 5′- TTGCCTTGACCCTGAAGCCCCC -3′		mouse
	Reverse: 5′- GGCACATCAGGTACGATCCAGGC -3′	175	
*kc*	Forward: 5′-ACTCAAGAATGGTCGCGAGG-3′		mouse
	Reverse: 5′-GTGCCATCAGAGCAGTCTGT-3′	123	
*mcp-1*	Forward: 5′- GGCTGGAGAGCTACAAGAGG-3′		mouse
	Reverse: 5′-GGTCAGCACAGACCTCTCTC-3′	93	
*cox-2*	Forward: 5′-GGGCTCAGCCAGGCAGCAAAT-3′		mouse
	Reverse: 5′- GCACTGTGTTTGGGGTGGGCT-3′	187	
*icam-1*	Forward: 5′- CTGGGCTTGGAGACTCAGTG-3′		mouse
	Reverse: 5′- CCACACTCTCCGGAAACGAA-3′	175	
*vcam-1*	Forward: 5′- CTGGGAAGCTGGAACGAAGT -3′		mouse
	Reverse: 5′- GCCAAACACTTGACCGTGAC-3′	115	
*tlr4*	Forward: 5′- TCAGCAAAGTCCCTGATGACATTCC-3′		mouse
	Reverse: 5′- AGAGGTGGTGTAAGCCATGCCA-3′	180	
*myd88*	Forward: 5′- GTCCATTGCCAGCGAGCTAA-3′		mouse
	Reverse: 5′- GGAGACAGGCTGAGTGCAAA-3′	120	
*vdr*	Forward: 5′- GCATCCAAAAGGTCATCGGC-3′		mouse
	Reverse: 5′- AGCGCAACATGATCACCTCA-3′	114	
*cyp27b1*	Forward: 5′- GCCGAGACTGGGATCAGATG-3′		mouse
	Reverse: 5′- TGATGCCCAGACGGCATATC-3′	115	
*cyp24a1*	Forward: 5′- CCCCAAGTGCAACAGAGACT-3′		mouse
	Reverse: 5′- CCGAGTTGTGAATGGCACAC-3′	153	
*18S*	Forward: 5′- CGGCTACCACATCCAAGGAA -3′		human
	Reverse: 5′- GCTGGAATTACCGCGGCT-3′	186	
*VDR*	Forward: 5′- ACCAGAAGCCTTTGGGTCTG-3′		human
	Reverse: 5′- CGTTCCGGTCAAAGTCTCCA-3′	146	
*TNF-α*	Forward: 5′- CACCACTTCGAAACCTGGGA-3′		human
	Reverse: 5′- TGTAGGCCCCAGTGAGTTCT-3′	105	
*IL-1β*	Forward: 5′- AACCTCTTCGAGGCACAAGG-3′		human
	Reverse: 5′- GGCGAGCTCAGGTACTTCTG-3′	107	
*IL-8*	Forward: 5′- ACCACCGGAAGGAACCATCT-3′		human
	Reverse: 5′- AGCACTCCTTGGCAAAACTG-3′	121	
*MCP-1*	Forward: 5′′- GATCTCAGTGCAGAGGCTCG-3′		human
	Reverse: 5′- TTTGCTTGTCCAGGTGGTCC-3′	155	
