# Supplementary material for: Vitamin D3 pretreatment regulates renal inflammatory responses during lipopolysaccharide-induced acute kidney injury
Source: Sci Rep. 2015 Dec 22;5:18687. doi: 10.1038/srep18687 (PMC4686931; doi:10.1038/srep18687)
Supplement: Supplementary Information [file srep18687-s1.doc]

**Supplementary information**

Vitamin D3 pretreatment regulates renal inflammatory responses during lipopolysaccharide-induced acute kidney injury

Shen Xu ^1,*^, Yuan-Hua Chen^2, 3, *^, Zhu-Xia Tan^1^, Dong-Dong Xie^1^, Cheng Zhang^2^, Zhi-Hui Zhang^2^, Hua Wang^2^, Hui Zhao^1^, De-Xin Yu^1, #^, De-Xiang Xu^2, #^

^1^The Second Affiliated Hospital, Anhui Medical University, Hefei, China;

^2^Department of Toxicology, Anhui Medical University, Hefei, China;

^3^Department of Histology and Embryology, Anhui Medical University, Hefei, China

**
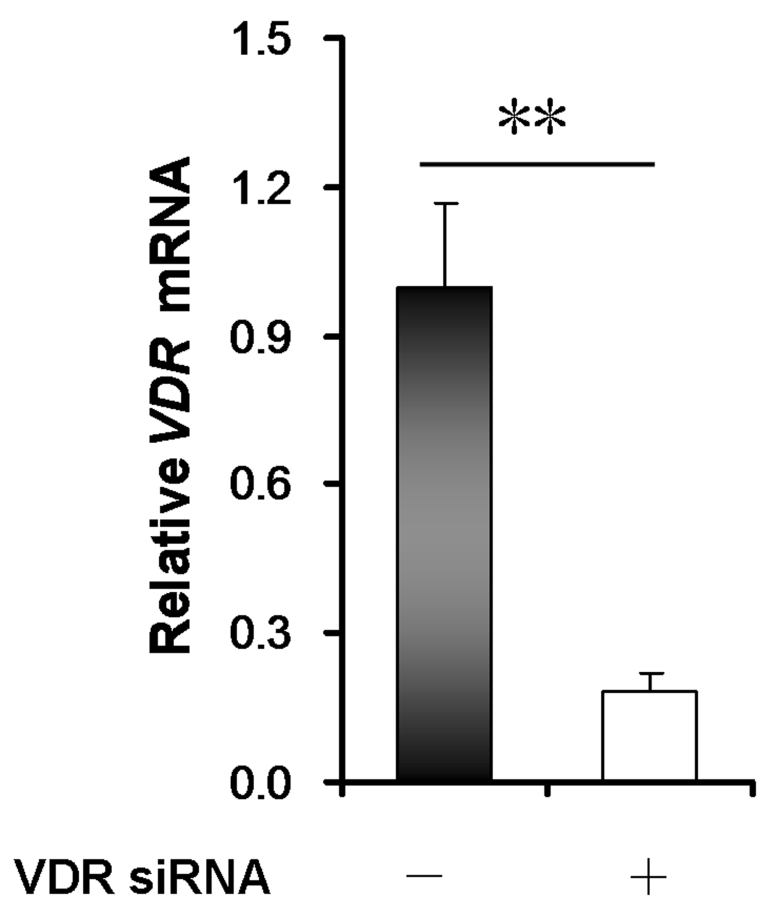
**

**Supplementary Figure 1.** Effects of VDR siRNA on the expression of *VDR* mRNA in human HK-2 cells. Human HK-2 cells were transfected with either pooled *VDR* siRNA (100 nM) or random siRNA (100 nM, as control) as Materials and Methods. *VDR* mRNA was measured using real-time RT-PCR. VDR siRNA (-) denotes the pretreatment with random siRNA as control. All data were expressed as means ± S.E.M (n=6). ** *P*<0.01.
